# Interindividual Variability in Stress Susceptibility: A Role for Epigenetic Mechanisms in PTSD

**DOI:** 10.3389/fpsyt.2013.00060

**Published:** 2013-06-26

**Authors:** Iva B. Zovkic, Jarrod P. Meadows, Garrett A. Kaas, J. David Sweatt

**Affiliations:** ^1^Department of Neurobiology, Evelyn F. McKnight Brain Institute, University of Alabama at Birmingham, Birmingham, AL, USA

**Keywords:** PTSD, epigenetics, stress, DNA methylation, chromatin, individual differences

## Abstract

Post-traumatic stress disorder (PTSD) is a psychiatric condition characterized by intrusive and persistent memories of a psychologically traumatic event that leads to significant functional and social impairment in affected individuals. The molecular bases underlying persistent outcomes of a transient traumatic event have remained elusive for many years, but recent studies in rodents have implicated epigenetic modifications of chromatin structure and DNA methylation as fundamental mechanisms for the induction and stabilization of fear memory. In addition to mediating adaptations to traumatic events that ultimately cause PTSD, epigenetic mechanisms are also involved in establishing individual differences in PTSD risk and resilience by mediating long-lasting effects of genes and early environment on adult function and behavior. In this review, we discuss the current evidence for epigenetic regulation of PTSD in human studies and in animal models and comment on ways in which these models can be expanded. In addition, we identify key outstanding questions in the study of epigenetic mechanisms of PTSD in the context of rapidly evolving technologies that are constantly updating and adjusting our understanding of epigenetic modifications and their functional roles. Finally, we discuss the potential application of epigenetic approaches in identifying markers of risk and resilience that can be utilized to promote early intervention and develop therapeutic strategies to combat PTSD after symptom onset.

Post-traumatic stress disorder (PTSD) is a devastating psychiatric condition that develops in the aftermath of a traumatic life event. It is characterized by persistent and intrusive memories that interfere with daily functioning, often to the point of physical and emotional disability (American Psychiatric Association, 2000). In recent years, epigenetic mechanisms in the central nervous system emerged as the long sought-after link between transient environmental stimuli, including trauma, and persistent changes in gene expression and behavior (Zovkic et al., 2013). Epigenetic mechanisms were initially implicated in behavior through their ability to shape stress- and mood-associated neural and behavioral outcomes caused by early life experiences (Champagne and Meaney, 2001; Meaney, 2001; Fish et al., 2004; Weaver et al., 2004a,b). Shortly after, the epigenetic mechanisms once thought to be stable across the lifespan were found to be dynamically regulated during learning and memory in adult rodents (Levenson and Sweatt, 2005, 2006; Chwang et al., 2006; Levenson et al., 2006; Lubin and Sweatt, 2007; Miller and Sweatt, 2007). This temporal duality implicated epigenetic mechanisms in shaping individual variation in risk and resilience in response to early life experiences and in mediating environmental triggers of psychiatric disorders in adulthood (McGowan et al., 2009; Labonte et al., 2012; Russo et al., 2012; Zovkic and Sweatt, 2013).

Vast individual differences in PTSD prevalence among people exposed to traumatic events (Kessler et al., 1995), the contribution of early life events to adult risk for psychopathology (Yehuda and Bierer, 2009; Kremen et al., 2012; Russo et al., 2012), and the ability of stressors to regulate gene expression (Cullinan et al., 1995) pointed to epigenetic involvement in PTSD before any direct evidence for a link was available (e.g., Yehuda and Bierer, 2009). Soon, human studies provided evidence linking epigenetics with PTSD, either as a consequence of trauma or as a mediator of gene-environment interactions in PTSD risk and resilience (e.g., Ressler et al., 2011; Skelton et al., 2012; Klengel et al., 2013; Norrholm et al., 2013). While comparatively few animal studies have directly investigated epigenetic involvement in PTSD, the related literature on the epigenetics of stress and fear conditioning provides important insights into the role of these mechanisms in establishing persistent fear in response to stressful stimuli (Zovkic and Sweatt, 2013). Thus, one of our goals is to review the relatively small literature that directly addresses the involvement of epigenetics in PTSD and put it into the broader context of epigenetics in stress and fear learning. Given that approximately one in eight individuals who experience traumatic life events develop PTSD (Kessler et al., 1995), we will place particular emphasis on individual differences in PTSD and the need for animal models to consider this variation in studies of risk and resilience. Finally, to encourage direct investigation of epigenetic mechanisms in animal models of PTSD, we will provide a brief technical overview of the rapidly evolving approaches for measuring DNA methylation that can serve as a launching pad for researchers interested in applying these tools to their own work. Overall, we will explore the idea that trauma induces long-lasting epigenetic changes that mediate PTSD-associated outcomes, and that the exact changes elicited are influenced by individual-specific epigenetic landscape on which the traumatic event occurs.

## Overview of Epigenetics in PTSD

Post-traumatic stress disorder is a multifaceted disorder that involves the deregulation of stress-responsive endocrine systems and of neurotransmitters and neuromodulators involved in arousal, depression, and anxiety (Baker et al., 2012). Although studies focused on identifying the relative contributions of these various systems have shaped the current understanding of the biological basis of PTSD, further progress will be driven by identifying common factors that drive and maintain their deregulation. Epigenetic modifications are emerging as one such factor because of their role in mediating gene expression for various components of the endocrine system, signaling molecules, and transcription factors (Champagne et al., 2006; Weaver et al., 2007; Chang et al., 2012; Russo et al., 2012; Klengel et al., 2013). In turn, epigenetic modifications are themselves regulated by upstream signaling cascades that converge to mediate epigenetic modifications in the nucleus, whereas existing epigenetic states alter the efficacy of the upstream signaling cascades on gene expression and behavior (Levenson et al., 2004; Fischer et al., 2007; Roozendaal et al., 2010; Graff and Tsai, 2011; Graff et al., 2011). In addition to providing an integrated perspective on psychiatric disorders, this bidirectionality offers promise for epigenetically based pharmacotherapies, which can reduce the threshold for, and amplify the response to, upstream signals (Graff and Tsai, 2011).

### Epigenetic mechanisms

DNA methylation initially gained notoriety for its role in the maintenance of cellular identity and heritable changes in gene expression throughout the cell cycle. DNA is initially methylated at the 5′ position of the cytosine–pyrimidine (5mC) ring by *de novo* DNA methyltransferase enzymes DNMT3a and 3b (Chiang et al., 1996; Turker, 1999; Bird, 2002; Cheng et al., 2010). This pattern of methylation is maintained throughout cell division by the maintenance DNMT1, which methylates cytosines opposite a methylated strand to allow for self-perpetuation of DNA methylation across cell divisions (Santos et al., 2005; Cheng et al., 2010). Given the energetic cost of removing a covalent 5mC bond and its role in the maintenance and propagation of cellular identity, 5mC was classically considered to be an irreversible mark. This notion has held largely true for the maintenance of cell-specific gene expression, but the classical view was challenged by evidence of active DNA demethylation in response to environmental stimuli in the brain, estrogen stimulation in cultured cells, and in muscle after exercise (Levenson et al., 2006; Miller and Sweatt, 2007; Kangaspeska et al., 2008; Metivier et al., 2008; Barres et al., 2012).

Indeed, 1.4% of measured CpGs (cytosines adjacent to guanines) in the granule cells in the dentate gyrus of the hippocampus exhibited altered DNA methylation in response to neuronal activation (Guo et al., 2011a), indicating that active DNA methylation and demethylation are a normal outcome of neuronal activity. Mechanisms of active DNA demethylation have been elusive, with limited success of early efforts to identify enzymes that remove 5mC directly. However, recent studies have provided a flurry of evidence suggesting that active demethylation is a stepwise process in which modifications of 5mC create a base mismatch that is replaced by an unmethylated cytosine through base excision repair (BER) (Wu and Zhang, 2010). Oxidation of 5mC to 5-hydroxymethylcytosine (5hmC) has emerged as a key step in active DNA demethylation in the brain and is accomplished by the ten-eleven translocation (TET) family of methyl-cytosine-dioxygenases (Tahiliani et al., 2009; Guo et al., 2011b; Ito et al., 2011). The resulting 5hmC is further modified by AID/APOBEC deaminases to yield 5 hydroxymethyluridine (5hmU) that is recognized by TDG and MBD4 to generate apurinic/apyrimidinic (AP) sites, which are cleaved by AP endonucleases and subjected to BER for replacement with an unmodified cytosine (5mC) (Ma et al., 2009a,b; Fritz and Papavasiliou, 2010; Bhutani et al., 2011; Guo et al., 2011b; Niehrs and Schafer, 2012; Grayson and Guidotti, 2013). Gadd45 proteins have also been implicated in this process, presumably by linking the AID/APOBEC deaminases with glycosylases to promote BER, which is particularly relevant for activity induced demethylation given the high sensitivity of Gadd45 to neural activity (Ma et al., 2009a,b). The much higher abundance (10-fold) of 5hmC in the rodent and human brain relative to other tissues (Kriaucionis and Heintz, 2009; Munzel et al., 2010; Jin et al., 2011; Szulwach et al., 2011), its increased levels over development (Munzel et al., 2010; Song et al., 2011b), and its regulation by neuronal activity (Guo et al., 2011b), suggest that this modification may be particularly relevant for cognitive function.

DNA methylation was initially identified as a mechanism for persistent repression of gene activity by the recruitment of co-repressor complexes (Karymov et al., 2001; Drewell et al., 2002) and the direct interference of 5mC with binding of transcriptional machinery (Iguchi-Ariga and Schaffner, 1989) to gene promoters. However, recent studies have shown that this is not always the case and that 5mC may at times be associated with gene *activation*. For example, although DNMTs are traditionally implicated in gene silencing (Chen et al., 2002), DNMT3a2 was recently associated with gene activation and improved cognitive function (Kotini et al., 2011) and *de novo* DNMTs may even be involved in DNA demethylation (Chen et al., 2012a). New evidence also suggests that the effect of DNA methylation on transcription may depend on the specific site on which the modification occurs, with 5mC at gene promoters and regulatory elements promoting gene repression and 5mC in gene bodies promoting gene activity (Hellman and Chess, 2007; Zilberman and Henikoff, 2007; Maunakea et al., 2010; Shenker and Flanagan, 2012). Although gene repression has been associated with the methylation of CpG islands in gene promoters, recent studies suggest that only 6.8% of CpGs are found in CpG islands (Rollins et al., 2006) and CpG islands are not ubiquitous to all human promoters (Deaton and Bird, 2011). In fact, newly identified CpG-island flanking regions termed CpG shores exhibit comparatively low CpG density and higher variability in methylation in cancer compared to CpG islands (Irizarry et al., 2009), implicating these sites in gene regulation. It is not clear to what extent these site-dependent functional differences are attributable to 5mC compared to 5hmC, since recent studies have demonstrated that 5hmC is depleted from promoters and intergenic regions and is enriched in gene bodies of actively transcribed cerebellar genes (Mellen et al., 2012). In contrast, a 5hmC peak that appeared 900 bp 5′ of the transcription start site was not correlated with transcription (Mellen et al., 2012), perhaps suggesting an alternative function. Of note, 5mC in the latter study was depleted from the bodies of actively transcribed genes and there was a tendency for a negative association between intragenic 5mC and gene expression in some cell types more than others (Mellen et al., 2012). Overall, however, intragenic 5hmC:5mC ratio was a better predictor of gene expression than 5mC or 5hmC alone (Mellen et al., 2012), pointing to the importance of considering both modifications in relation to transcription in different cell types. More broadly, these data indicate that the role for DNA methylation is more heterogeneous than initially suspected and that further studies will be required to fully understand the relationship between DNA methylation and transcription.

Many effects of DNA methylation are exerted through interactions with DNA-binding factors that recruit co-activators or co-repressors to mediate gene transcription and post-translational modifications (PTMs) of histones (Strahl and Allis, 2000; Ooi et al., 2007; Borrelli et al., 2008). DNA is packaged into nucleosomes, which are the building blocks of chromatin and are composed of ∼147 bp of DNA wrapped around an octamer of 2 each of histones H2A, H2B, H3, and H4 (Quina et al., 2006). Histones are critical regulators of DNA accessibility and chromatin compression or openness is determined in part by PTMs of histones (Strahl and Allis, 2000). Histones contain protruding tails that can be modified in a number of ways, most notably through acetylation, methylation, and phosphorylation (Strahl and Allis, 2000). As with DNA methylation, the pattern of histone modifications appears to differ based on the genomic region of interest, notably between introns and exons and around the transription start site (TSS) (Lieb and Clarke, 2005; Zilberman and Henikoff, 2007; Choi et al., 2010; Huff et al., 2010).

Of these various chromatin modifications, histone acetylation has received the most attention and is typically involved in the activation of gene expression (Mujtaba et al., 2007), whereas histone methylation can be involved in either activation or repression (Nakayama et al., 2001; Peters and Schubeler, 2005). Histone acetylation is catalyzed by histone acetyltransferases (HATs) and the reverse deacetylation reaction is performed by a large number of histone deacetylases (HDACs) (Hebbes et al., 1988; Wade, 2001). The binding of these enzymes is influenced by DNA methylation and associated methyl-CpG binding proteins, particularly MeCP2, which recruits HDACs to gene promoters to facilitate histone deacetylation (Lorincz et al., 2004; Liu et al., 2005). Recent evidence from the model plant *Arabidopsis thaliana* and from treatment with the HDAC inhibitor (HDACi) valproic acid (VPA) suggests that the reverse reaction may also be true, with HATs participating in DNA demethylation (Tremolizzo et al., 2002, 2005a,b; Detich et al., 2003; Qian et al., 2012). Indeed, the methyl-CpG binding protein MeCP2 interacts with CREB to promote histone acetylation (Chahrour et al., 2008), although it is not clear whether this relationship occurs through MeCP2 interactions with 5hmC and 5mC (Mellen et al., 2012).

MeCP2 binds to both 5mC and 5hmC in the cerebellum, but binding at 5hmC was associated with accessible chromatin and gene activation, whereas binding at 5mC was associated with closed chromatin and gene repression (Mellen et al., 2012). These effects were mediated by different residues on MeCP2, with a mutation in R133C preferentially altering binding to 5hmC compared to 5mC (Mellen et al., 2012). Other have also reported chromatin-regulating effects of neurally expressed MeCP2, with a weak association of MeCP2 with open chromatin and a strong association with closed chromatin (Thambirajah et al., 2012). Overall, there is some overlap in DNA-binding factors that interact with 5mC and 5hmC, but each modification also has unique interacting partners and different affinities for certain MBDs and other DNA-binding proteins (Hashimoto et al., 2012; Spruijt et al., 2013). These data suggest that 5hmC may be a transcriptional regulator in its own right rather than having a sole function as an in intermediary in active DNA demethylation.

The rapid advancements in techniques for detecting 5mC and 5hmC (see **Box 1**) and the application of whole-genome sequencing approaches have led to an unprecedented rate of growth in our understanding of epigenetic mechanisms in the regulation of gene expression. The wealth of new information has challenged many of the classical views of epigenetics in general and DNA methylation in particular, with many important implications for interpreting past and future data in the field. The most direct lesson from recent studies is that epigenetic modifications have diverse roles in gene regulation and that their effect on gene expression cannot be directly inferred from examining epigenetic modifications alone.

Box 1 **Review of tools for measurement of hydroxymethylation.**

**Global 5hmC analysis**

Several options exist for the global quantification of nucleotide variants implicated in epigenetic regulation, which include 5-methylcytosine (5mC), 5-hydroxymethylcytosine (5hmC), 5-formylcytosine (5fC), and 5-carboxylcytosine (5caC), respectively. Currently, various antibodies raised against these epigenetic markers are available (Inoue et al., 2011; Ito et al., 2011) and have been employed in combination with either an enzyme-linked immunosorbent assay (ELISA) or dot-blot analysis for global detection (Koh et al., 2011; Li and Liu, 2011). In addition, some have taken advantage of the enzyme T4 phage β-glucosyltransferase (T4-GβT) and its ability to specifically add glucose to 5hmC to generate β-glucosyl-5-hmC (5gmC) (Terragni et al., 2012). Using this process, a radio-labeled glucose can then be transferred to 5hmC for sensitive and accurate detection of its global levels (Lian et al., 2012).

Another strategy to identify nucleotide modifications involves the use of thin layer chromatography (TLC) coupled with radio labeling. This approach provides a high level of sensitivity and was implemented by several labs leading to the initial discoveries of both 5hmC (Kriaucionis and Heintz, 2009; Tahiliani et al., 2009) and 5caC (He et al., 2011; Ito et al., 2011), respectively. However, of all the methods mentioned above, the gold standard remains the use of liquid chromatography (LC) followed by MS to resolve and precisely measure nucleosides containing these epigenetic marks (Globisch et al., 2010; Munzel et al., 2010; Ito et al., 2011).

**Loci specific analysis and 5hmC enrichment**

**hMeDIP:** A number of methods are now available for the measurement of 5hmC levels at the resolution of individual genomic loci. The first, hydroxymethyl-DNA immunoprecipitation (hMeDIP) requires the enrichment of 5hmC-containing DNA, typically with an antibody raised against 5hmC or its sodium bisulfite converted derivative, 5-methylsulfonate (CMS) (Pastor et al., 2011). Alternatively, enrichment can be achieved through T4-GβT-mediated conversion of 5hmC to 5gmC, followed by incubation with J-binding protein-1 (JBP-1); a protein shown to have a strong affinity for the modified base (Robertson et al., 2011). Likewise, some groups have gone on to process 5gmC even further through oxidation and biotinlyation steps (Pastor et al., 2011) (GLIB) or with selective chemical labeling (Song et al., 2011a) (hMe-Seal), allowing for the enrichment of biotinylated 5hmC residues using streptavidin beads. Using either the GLIB or hMe-Seal strategies allow for improved hMeDIP fidelity as well as the enrichment of genomic regions containing low amounts of 5hmC.

**Restriction enzyme digestion:** Recently, a number of restriction enzymes whose activity is blocked either by 5hmC itself or after 5hmC glycosylation have been characterized (Zheng et al., 2010; Cohen-Karni et al., 2011; Davis and Vaisvila, 2011; Kinney et al., 2011; Song et al., 2011b; Szwagierczak et al., 2011; Wang et al., 2011; Xu et al., 2011). The latter of these enzymes have been termed glucosyl-5-hydroxymethylcytosine sensitive restriction endonucleases (GSREs) and several companies now offer commercial kits to investigators. Due to their unique properties, these enzymes can be used to identify specific 5hmc-containing loci provided the region of interest contains the enzymes recognition site. Difficulties arising due to lack of compatible restriction endonuclease recognition sites in genomic regions of interest may be overcome using several different GRSEs. This technique has been further extended using several GSREs in combination, which enables the determination of both 5hmC and 5mC content at the same genomic location (Davis and Vaisvila, 2011).

Both techniques outlined here are amenable for analysis using quantitative PCR analysis for 5hmC detection at individual loci or for whole-genome profiling, microarray analysis, and next generation sequencing.

**Single-base resolution sequencing**

Although hMeDIP and GRSE analysis can both be useful tools for the analysis of 5hmC distribution on a genome-wide scale, they are limited in their resolution due to DNA fragmentation size and capture technology. To overcome these limitations, several groups have developed techniques to achieve the sequencing of 5hmC residues at a single-base resolution. The first involves the use of third-generation sequencing technology called single-molecule, real-time (SMRT) sequencing which monitors the incorporation of individual nucleotides by DNA polymerase in real time (Eid et al., 2009) and can detect DNA base modifications like 5mC and 5hmC compared to unmodified cytosine due to changes in DNA polymerase kinetics (Flusberg et al., 2010). This technique has now been combined with hMe-Seal to chemically label 5hmC in an effort to distinguish it further from 5hmC and C during DNA polymerization (Song et al., 2012). A second method termed oxidative bisulfite sequencing (oxBS-Seq) involves comparison of a traditional bisulfite sequenced sample (BS-seq) to that which has undergone oxidative conversion of 5hmC to 5fC, prior to bisulfite conversion (Booth et al., 2012). While BS-seq can convert both 5mC and 5hmC to cytosines (Cs), oxBS-Seq only allows for conversion of 5mC sites to Cs. Thus, the amount of 5hmC present at a particular nucleotide position can then be determined by subtraction of the oxBS-Seq sample from that of the BS-Seq sample. Finally, Tet-assisted bisulfite sequencing (TAB-seq) is a technique which combines the protection of 5hmC by T4-GBT glycosylation with Tet-mediated oxidation of 5mCs to 5caCs (Yu et al., 2012). After bisulfite conversion all 5caCs are converted to Ts, leaving only protected 5hmC residues as Cs, resulting in a straight-forward interpretation of 5hmC data at a base-by-base resolution.

Although we have focused heavily on DNA methylation, even the typically activating histone acetylation mark has been found on bivalent promoters that can be either activated or repressed (Lin et al., 2007; Vastenhouw and Schier, 2012) and HATs and HDACs tend to colocalize on the same genomic sites (Peserico and Simone, 2011). Similarly, in addition to their role in oxidation of 5mC to 5hmC, TET proteins may have additional regulatory roles, as exemplified by TET1 interaction with a repressive complex that might have a role in gene repression independent of its role in DNA demethylation (Bhutani et al., 2011; Ito et al., 2011; Wu and Zhang, 2011; Williams et al., 2012).

Another critical point is that many of the earlier studies did not distinguish between 5mC and 5hmC, making it difficult to draw inferences regarding the relative contribution of each. Although many approaches have been developed to differentiate the two, many techniques still encompass both modifications (see **Box 1**). Finally, growing evidence for distinct functions of DNA methylation at different genomic sites indicates that the common approach of using a single primer per gene may not be sufficient to detect changes in DNA methylation, or that the direction of the change may depend on the region studied (i.e., intragenic vs. promoter; CpG island vs. CpG-island shore). One solution to this approach is to do whole-genome sequencing, although a cost effective alternative is to utilize multiple primers to represent different regions relevant for detecting functional effects. An additional confound in neurobiology is the difficulty in distinguishing between epigenetic modifications occurring in neurons and glia, which will require more wide-spread use of cell sorting approaches and neuron-specific labels to improve our understanding of epigenetic changes in these distinct cell types.

## Animal Models

Well-designed animal models are critical for uncovering epigenetic mechanisms of PTSD by allowing for the control of the temporal parameters needed to understand predisposition, onset, maintenance, and treatment of the disorder. An appropriate animal model must ensure face and etiological validity by mimicking the conditions of the disorder seen in human patients, which primarily involves exposure to a traumatic event in the case of PTSD (van der Staay et al., 2009). The traumatic event has been modeled in a number of ways, including exposure to an aversive shock in fear conditioning, exposure to a predator, social defeat, restraint, or underwater holding (Adamec and Shallow, 1993; Richter-Levin, 1998; Cohen et al., 2000a,b; Siegmund and Wotjak, 2007; Zoladz et al., 2008; Zovkic and Sweatt, 2013). Given the association between trauma and symptom severity in human patients (Koenen and Uddin, 2010; Uddin et al., 2010), stimuli that can deliver graded levels of trauma are preferred in an animal model (Siegmund and Wotjak, 2007). Shock delivery in fear conditioning is particularly amenable to manipulating gradations in stimulus intensity and duration (Siegmund and Wotjak, 2007), although different degrees of trauma intensity can also be achieved in predator exposure models by exposing rodents to cat odor alone, a caged cat, or to a direct encounter with a cat (Zoladz et al., 2008; Goswami et al., 2012). The etiological validity of these models can be further improved by combining the traumatic experience with additional risk factors that further enhance vulnerability to PTSD, such as the lack of social support and persistent exposure to chronic mild stress (Zoladz et al., 2008). In one example, the number of rodents expressing PTSD symptoms was increased by combining predator odor exposure with 31 days of social instability stress in an effort to mimic the lack of social support seen in individuals vulnerable to PTSD (Zoladz et al., 2008).

As an extension of the face validity criterion, animal models must include dependent variables that parallel the diverse symptoms of PTSD in order to generate testable predictions for research and treatment in human patients (van der Staay et al., 2009). PTSD patients exhibit a wide range of symptoms, including hyperarousal, increased startle, emotional blunting, and social withdrawal, which can be included as outcome variables in response to a variety of traumatic stimuli (Siegmund and Wotjak, 2007; Uddin et al., 2010; Skelton et al., 2012). These symptoms are induced by fear conditioning and predator exposure models of PTSD and are typically assessed after a prolonged stress-free period (Zovkic and Sweatt, 2013). For example, fear conditioning can be used to assess intensity-dependent freezing upon re-exposure to the training context to test for the expression of associative fear and upon exposure to a novel, neutral tone to provide an index of non-associative fear sensitization, hyperarousal, and startle (Siegmund and Wotjak, 2007). Importantly, the expression of sensitized fear to a novel auditory cue was found to increase with time after shock exposure (Siegmund and Wotjak, 2007), which is particularly important in light of the proposed revisions to DSM-V that emphasize delayed symptom onset over the initial fear response (Resick and Miller, 2009; Friedman et al., 2011a,b). Indeed, many patients who are diagnosed with PTSD show little to no emotional response upon initial exposure to trauma, with symptoms developing only with the passage of time (Shalev et al., 2000; Griffin, 2008; Friedman et al., 2011a,b). Associative fear is particularly amenable to delayed behavioral assessment (Miller et al., 2010) and has the advantage of modeling the re-experiencing of fear in PTSD patients by presenting animals with an aversive cue or context without the need for re-exposure to the traumatic stimulus (i.e., footshock) (Zovkic and Sweatt, 2013).

### Models of predisposition to PTSD

A difficulty with PTSD studies in humans is the poor ability to distinguish between pre-existing risk factors and trauma-induced outcomes. Co-twin studies, in which only one twin has experienced trauma, found that certain parameters that were thought to be caused by trauma may actually be pre-existing risk factors for PTSD, including impaired cognitive function and reduced hippocampal volume (Kremen et al., 2012). It can also be difficult to separate out epigenetic outcomes from epigenetic risk factors in non-controlled studies of human patients, although cumulative effects of trauma on DNA methylation of immune system genes suggest that traumatic experiences are key drivers of epigenetic outcomes in this scenario (Uddin et al., 2010).

Animal models can be particularly valuable in this regard by evaluating temporal parameters of variables identified in human studies to determine their relative role in conferring risk for PTSD or outcomes of trauma. An evaluation of individual differences is a key component for the success of such models (Yehuda and Bierer, 2009). The emphasis of animal models of PTSD tends to be on the stress exposure (induction) and the associated depressive and anxious phenotypes (read out), which are valuable tools for defining the cognitive, molecular, and neuroanatomical outcomes of stress exposure, but are less productive in explaining the relatively low incidence of persistent psychopathology in response to trauma (Yehuda et al., 2006). Individual differences in rodents can be investigated by classifying animals according to natural variation in behavior, by utilizing genetic predictors of risk and selective breeding strategies based on traits associated with risk and resilience (Scharf and Schmidt, 2012). Cohen and Zohar (2004) developed a model of classifying rodents according to natural variation in responses to predator exposure, in which ∼22% of the rats meet the PTSD criteria based on behavioral, endocrine, and sympathetic markers. The *ad hoc* classification system used in these studies is extremely useful for understanding long-term adaptations in behavioral and molecular systems that distinguish vulnerable and resilient individuals after trauma, but such models do not provide any information on the source of vulnerability or resilience before trauma (Siegmund and Wotjak, 2007; Zovkic and Sweatt, 2013). Selective breeding of vulnerable and resilient mice can help circumvent this problem by allowing for the assessment of pre-existing differences prior to trauma (Siegmund and Wotjak, 2007). For example, strain differences have been noted between PTSD vulnerable B6N and the resilient B6JOla mice, with B6N exhibiting blunted affect and reduced sociability in the absence of any exposure to trauma (Siegmund and Wotjak, 2007). Genetic mouse models can also be used to evaluate the role of risk genes identified in human studies and these models can be combined with developmental stressors to probe for gene-environment interactions at distinct stages of development.

History of stress exposure, particularly in early life, is another essential factor in predisposing individuals to PTSD in adulthood (Scharf and Schmidt, 2012). Although most rodent studies of early environment do not relate behavioral and biochemical outcomes to PTSD directly, their relevance to PTSD has been widely recognized because of the emphasis on the stress-responsive neural circuitry and the related behavioral outcomes (Yehuda and Bierer, 2009).

A widely studied model utilizes variation in maternal licking and grooming (LG) and arched back nursing (ABN) of pups as an index of early life experience. In this model, the offspring of high LG-ABN mothers are less anxious and responsive to stressors compared to the offspring of low LG-ABN dams (Weaver, 2009). However, the extent to which one maternal style is “better” than the other is not clear, as the offspring of high and low LG mothers may each be better adapted to different environmental circumstances (Bagot et al., 2009). However, the offspring of low LG mothers are phenotypically similar to the pups that underwent maternal separation stress (≥3 h separation) and high LG offspring are similar to those who underwent a milder brief separation (15 min), often referred to as handling, in the first 7–14 days of life (Anisman et al., 1998; Francis et al., 2002; Wilber and Wellman, 2009), indicating that low LG-ABN is akin to a more stressful upbringing. Utilization of maternal maltreatment models with an unambiguous stress exposure in early life (Roth and Sweatt, 2011) will be particularly useful in clarifying the association between stressful upbringing and behavior. Moreover, combining these early life models with adult models of PTSD (Cohen and Zohar, 2004) can provide critical information regarding the interaction between early life experience and the later risk and resilience for PTSD.

## PTSD and the HPA Axis

### The HPA axis

Many studies of individual differences in risk and resilience have focused on the hypothalamic-pituitary-adrenal (HPA) axis because of its central role in adaptation to stress. The diagnosis of PTSD requires that symptom onset be preceded by a traumatic event and PTSD was recently reclassified as a traumatic and stress-related disorder instead of an anxiety disorder in the newly released DSM-V (Friedman et al., 2011a,b). Stressful encounters induce the release of corticotropin releasing hormone (CRH) from the hypothalamus into the pituitary gland to stimulate the release of adrenocorticotropin hormone (ACTH) and activate the release of glucocorticoids (GCs; cortisol in people, corticosterone in rodents) from the adrenals (Novak et al., 2013). The acute GC response allows individuals to deal with stressors by mobilizing resources, adjusting heart rate, and regulating immune function in the short term, whereas deregulation of GC release over prolonged time periods is detrimental to health, mood, and cognition (Sorrells and Sapolsky, 2007).

GC receptors (GR) are found throughout the brain and are particularly abundant in the hippocampus, where they influence memory formation, cognitive function, and initiate negative feedback to reinstate baseline GC levels after stress (Novak et al., 2013). The HPA axis is a critical regulator of the response to trauma in adults and the deregulation of this response has been implicated in the development of PTSD (Yehuda et al., 2004, 2009). PTSD patients generally exhibit reduced levels of GCs, increased negative feedback, abnormal GR expression, and higher levels of CRH compared to non-traumatized controls (Yehuda et al., 2002; Rohleder et al., 2004; de Kloet et al., 2008), indicating that a blunted GC response to stress may be a risk factor for PTSD (Yehuda and McFarlane, 1995; Yehuda and LeDoux, 2007).

Exposure to GCs stimulates a range of epigenetic modifications that are though to be mediated by the GR (Roozendaal et al., 2010; Yang et al., 2012). The GR is a transcriptional regulator that translocates to the nucleus upon ligand binding, where it activates or represses transcription through actions on the glucocorticoid response element (GRE) (Cairns et al., 1991). According to some estimates, GCs regulate between 1000 and 2000 genes in cortical neurons (Kino, 2007; Sun et al., 2008) and chronic stressors regulate gene expression and histone modifications in neural regions associated with cognitive function, affect, and motivation (Tsankova et al., 2004, 2006; Covington et al., 2011a,b). Effects of GR on transcription are closely tied to epigenetic regulation, as exposure to stress or treatment with glucocorticoids results in the persistent and gene-specific demethylation of GREs in peripherally derived human cells and the rodent brain (Unternaehrer et al., 2012; Yang et al., 2012; Klengel et al., 2013). In addition, exposure to stress in early life alters the expression of epigenetic regulators themselves, as evidenced by increased levels of DNMT3a in the placenta and the brain of pups of stressed mothers (Jensen Pena et al., 2012). Moreover, maternal maltreatment and natural variation in maternal care induce modifications of DNA methylation and expression of target genes that persist into adulthood in the rodent brain (Weaver et al., 2002; Zhang et al., 2006; Roth and Sweatt, 2011). In addition to the direct effects of GR in the nucleus, the GR mediates histone acetylation at target genes indirectly through the activation of the PKA-pCREB/CBP pathway via its membrane-bound receptor (Roozendaal et al., 2010). Overall, these studies demonstrate the ability of stressors to regulate epigenetic modifications, presumably through the actions of GCs on the GR receptor, although other potential pathways, such as sympathetic and noradrenergic responses, cannot be ruled out (Geracioti et al., 2001; Bierer et al., 2006; Videlock et al., 2008).

In addition to regulating epigenetic modifications, the HPA axis is itself epigenetically regulated, particularly during development, when the GR, CRH, and the GC inactivating enzyme *HSD11B2* exhibit persistent alterations in DNA methylation and gene expression in response to maternal stress and natural variation in maternal behavior (Weaver, 2009; Roth and Sweatt, 2011; Jensen Pena et al., 2012). It has been shown, for example, that maternal deprivation between postnatal days 2 and 13 leads to hypomethylation of the cyclic AMP-response element (CRE) in the CRH promoter in the hypothalamus (Chen et al., 2012b), which is particularly relevant given the higher levels of CRH in patients with PTSD (de Kloet et al., 2008). Similarly, offspring of low LG mothers exhibit higher GR methylation, reduced GR expression, and higher reactivity to stress and anxiety in adulthood compared to the offspring of high LG mothers (Weaver, 2009). This effect is established in the first 7 days of life, when high levels of LG induce increased NGFI-1 expression, an upregulation of histone acetylation, and a decrease in DNA methylation at the NGFI-1 binding site of the GR promoter (Weaver et al., 2002, 2004a; Weaver, 2009). Extensive individual differences in DNA methylation of the GR promoter have been reported in people and include increased methylation of the NGFI-1 binding site on the GR promoter and decreased GR expression in the hippocampus of suicide victims with a history of childhood abuse compared to non-abused suicide victims or controls (McGowan et al., 2009).

It thus appears that elevated activity of DNA-binding molecules, such as GR and NGFI-I, may regulate binding to their respective response elements by reducing DNA methylation at the appropriate sites. This relatively well characterized system of maternal regulation of GR expression illustrates the close bidirectional relationship between epigenetic mechanisms and upstream signaling cascades. Moreover, the observation that persistent epigenetic changes are established in the first 7 days of life (Szyf et al., 2007) suggests that long-lasting effects of stress, maternal behavior, and GC exposure are amplified during sensitive periods of development, although the GR promoter is also subject to epigenetic regulation in adulthood (Witzmann et al., 2012).

Depending on the type of maternal care, epigenetic programing of GC release and GR expression may promote vulnerable or resilient phenotypes in adulthood (Dudley et al., 2011). On the surface, some parallels can be drawn between high LG mothers and the children of Holocaust survivors who had lower levels of GCs and rated their mothers as overprotective (Yehuda and Bierer, 2009). Based on such evidence, Yehuda and Bierer (2008) argued that reduced GC levels in high LG offspring parallel reduced cortisol, increased lymphocyte GR expression, and increased GR sensitivity seen in human PTSD patients (Yehuda et al., 1998, 2002; Yehuda, 2002a,b), although others interpret the reduced cortisol response to stress as protective (Barha et al., 2007). However, there are also reports of reduced levels of GR in leukocytes of PTSD patients (de Kloet et al., 2007), which parallels the reduced levels of GR density seen in low LG offspring. Furthermore, prenatal maternal depression/anxiety leads to increased methylation within the GR promoter at a predicted NGFI-A binding site in infants (Oberlander et al., 2008), likely leading to an overall reduction in GR expression. It is difficult to draw comparisons based on circulating GC and peripheral GR levels alone and studies of GR density in post-mortem brains of PTSD patients are needed to draw clearer parallels. The contention that the offspring of high LG mothers exhibit parallel HPA axis activity to PTSD patients (Yehuda and Bierer, 2009) is consistent with the view that some stress exposure at distinct stages of development confers resilience to PTSD in adults through “stress inoculation” (Ricon et al., 2012). Given the phenotypic similarities between outcomes associated with maternal separation stress and low LG offspring (Anisman et al., 1998; Francis et al., 2002; Wilber and Wellman, 2009), the concept of protective effects of “stress inoculation” (Ricon et al., 2012), and the adaptive function of an appropriate GC response to stress (Yehuda and McEwen, 2004), some have suggested that low LG offspring are actually better adapted to deal with stressful environments than high LG offspring (Bagot et al., 2009).

## Gene × Environment × Epigenome Interactions

Individual differences in adult risk and resilience to PTSD are shaped by a combination of genetic and environmental factors. Although genes and environment can produce shifts in risk and resilience on their own, the strongest phenotypic effects are associated with gene-environment interactions that are mediated by epigenetic modifications (Caspi and Moffitt, 2006; Chertkow-Deutsher et al., 2010; Dudley et al., 2011; Russo et al., 2012; Klengel et al., 2013; Zovkic and Sweatt, 2013). Environmental factors are particularly influential during early life, when they interact with epigenetic mechanisms to lastingly alter the expression of a broad range of genes (Sundermann et al., 2012, 2013).

Studies of stress exposure in rodent strains with differential susceptibility to risk and resilience have reported distinct epigenetic and behavioral outcomes, although the specific genes involved in mediating these effects have not been identified. For example, vulnerable BALB/c (BALB) mice exhibited differences in H3 acetylation and DNA methylation of the glial cell-derived neurotrophic factor (GDNF) in the nucleus accumbens compared to the resilient C57BL/6 (B6) mice (Uchida et al., 2011). In addition, BALB mice showed a specific increase in MeCP2-HDAC2 association and B6 showed an increase in MeCP2-CREB association at the GDNF promoter, which were associated with increased and decreased GDNF expression in B6 and BALB mice, respectively. Vulnerability and resilience were also associated with opposite changes in DNA methylation of the same genes (Elliott et al., 2010), and with different levels of intronic DNA methylation and expression of *dlgap2*, a gene coding for PSD95 (Chertkow-Deutsher et al., 2010). In contrast, rodents classified as vulnerable or resilient based on behavioral responses to acute unavoidable stress exhibited differential regulation of distinct genes, with upregulation of neuroendocrine-related genes, growth hormone, and prolactin genes in vulnerable rats, and the downregulation of interferon-β and leukemia inhibitory factor in the frontal cortex of resilient rats (Benatti et al., 2012). Furthermore, Oh et al. (2013) found that an adverse maternal environment in rodents was associated with differentially methylated sites that clustered in the bodies of genes associated with cell adhesion and neurotransmitter receptors in the hippocampus. Finally, a study investigating the effects of environmental stress in non-human primates found an association between increased stress reactivity and higher overall methylation (Kinnally et al., 2011), which led them to speculate that the overall increase in methylation constrained plasticity through gene repression. However, the degree to which this hypothesis will hold true in light of evidence for an activating role of DNA methylation remains to be determined. Overall, these studies demonstrate that similar environmental experiences can produce extremely different outcomes through epigenetic interactions with individual-specific factors that mediate vulnerability and resilience.

### The HPA axis

Studies of human patients have found genotype-specific effects of early environment on adult risk for PTSD to be mediated by epigenetic modifications of genes associated with the HPA axis (Koenen and Uddin, 2010; Ressler et al., 2011; Klengel et al., 2013). *Fkbp5* is a negative regulator of GR activity that has received much attention in this regard, in part because of reduced *Fkbp5* and cortisol levels in the patient population (Yehuda and Bierer, 2009; Yehuda et al., 2009; Koenen and Uddin, 2010; Klengel et al., 2013). Single nucleotide polymorphisms (SNPs) in the *Fkbp5* gene are associated with predisposition to PTSD, wherein the same allele confers either risk or resilience in the presence or absence of childhood adversity, respectively (Xie et al., 2010). Specifically, SNPs around the GRE in the human *Fkbp5* gene increased risk for PTSD only in individuals who experienced childhood trauma (Binder et al., 2008; Yehuda et al., 2009). This effect was epigenetically mediated, in that GC exposure was associated with greater *Fkbp5* GRE demethylation and higher *Fkbp5* expression in carriers of the risk compared with the protective allele (Klengel et al., 2013). These data are in contrast to reports of reduced *Fkbp5* expression in PTSD patients (Yehuda et al., 2009), but the discrepancy may in part be explained by variability across peripherally derived cell types and the sorting of data according to genetic variation.

PAC1R, a receptor for PACAP (pituitary adenylate cyclase-activating peptide), is another gene involved in stress responsiveness that has been implicated in PTSD (Dias and Ressler, 2013). PACAP is elevated in the blood of female, but not male, patients with PTSD and an SNP in the estrogen response element of the PAC1R gene interacts with trauma to mediate risk for PTSD in affected individuals (Ressler et al., 2011). Moreover, DNA methylation of the PAC1R gene is positively associated with PTSD (Ressler et al., 2011), again emphasizing the role of epigenetic factors as mediators of gene-environment associations. These data provide critical insights into the factors involved in establishing vulnerable phenotypes during development, but comparisons of epigenetic responses in vulnerable and resilient adult rodents are required to understand how traumatic experiences produce different outcomes in those individuals.

### Synaptic plasticity and neurotransmitter genes

In addition to the HPA axis, gene-environment-epigenome interactions have been reported for neuromodulators, neurotransmitters, and other molecules relevant for synaptic function and plasticity. For example, the serotonergic system is of clear relevance to PTSD given its well documented, albeit complex role in the regulation of emotional circuitry and fear learning (Rainnie, 1999; Koponen et al., 2005; Christianson et al., 2010), which has prompted an interest in polymorphisms of the serotonin transporter gene *SLC6A4*. A recent meta-analysis found that a short allele with an SNP in the 5-HTT promoter was associated with reduced transcriptional efficiency and increased sensitivity to stress (Karg et al., 2011). As with *Fkbp5*, association of the risk allele with PTSD occurs primarily through interactions with environmental experiences (Kilpatrick et al., 2007; Koenen et al., 2009; Xie et al., 2009), although some discrepancies have been reported (Mellman et al., 2009; Koenen et al., 2011). Epigenetic mediation of gene-environment interactions in PTSD has also been reported for other genes related to synaptic function, such as genes coding for Neuropeptide Y (NPY), BDNF, and molecules involved in the noradrenergic and dopaminergic systems (Boulle et al., 2012; Russo et al., 2012; Skelton et al., 2012; Wu et al., 2013).

In contrast to *Fkpb5* and *SLC6A4*, carriers of the 9R allele of the dopamine transporter gene *SLC6A3* were at greater risk of developing PTSD compared to 10R/10R allele carriers irrespective of environmental factors (Chang et al., 2012), highlighting the independent contribution of certain genes and the need to consider environmental interactions on a gene-by-gene basis. Furthermore, the risk for PTSD was highest in 9R allele carriers who also had high promoter methylation (Chang et al., 2012), indicating that epigenetic factors may mediate the contribution of genetic risk factors, irrespective of environmental factors.

It is important to note that the studies discussed thus far have primarily utilized a candidate-gene approach that allows for a thorough assessment of polymorphisms in a single gene, but is not suited for identifying novel genetic candidates involved in mediating risk and resilience. A recent genome-wide association study (GWAS) of trauma-exposed individuals identified a single polymorphism in the gene encoding the retinoic acid orphan receptor A (RORA), to be associated with PTSD after applying stringent genome-wide significance criteria (Logue et al., 2012). The high-risk SNP was associated with increased risk for PTSD in response to lower levels of trauma compared to individuals with the protective allele. RORA is a member of the NR1 subfamily of nuclear hormone receptors with neuroprotective functions (Jolly et al., 2011), which led the authors to hypothesize that genetically mediated differences in RORA levels are associated with an impaired stress response (Logue et al., 2012). Studies such as this will be critical for identifying novel candidates for PTSD risk and resilience in distinct populations.

### Epigenetic mediation of risk and resilience

Although genetic factors are essential mediators of risk and resilience, epigenetic mechanisms also mediate the effects of severe trauma independently of predisposing factors (Chang et al., 2012). Indeed, the extent of epigenetic modifications observed in patients is amplified by the severity and the number of traumatic events experienced (Uddin et al., 2010; Koenen et al., 2011) and distinct methylation profiles of the serotonin transporter gene are differentially associated with risk and resilience even after controlling for genotype (Koenen et al., 2011). Furthermore, observations of altered DNA methylation in response to childhood abuse and bullying (Ouellet-Morin et al., 2012) indicate that epigenetic modifications can predispose individuals to risk or resilience over and above the variability induced by genetic factors, as found with maternal behavior in rodents. In fact, the sensitive period for establishing lasting effects of stress appears to extend beyond early life into juvenile and adolescent periods of development (McCormick and Mathews, 2010; Dudley et al., 2011), as evidenced by site-specific demethylation of *fkbp5* and reduced expression of DNMT1 in the mouse dentate gyrus of juvenile pups that were given 4 weeks of corticosterone treatment (Yang et al., 2012).

Although individual differences in PTSD risk are well established, relatively few studies have evaluated the specific mechanisms that underlie resilience. It is not yet clear whether resilience reflects the addition of a protective factor or the absence/reduction of a vulnerability factor (Russo et al., 2012). Studies of gene expression have found higher blood levels of NPY in resilient Special Forces soldiers (Morgan et al., 2000, 2002) and higher levels of c-Fos, FosB, and ΔFosB in resilient mice (Covington et al., 2010; Lehmann and Herkenham, 2011; Adamec et al., 2012), although genome-wide studies have reported altered expression in a comparable number of genes in resilient and vulnerable populations (Benatti et al., 2012). Some evidence suggests that risk and resilience are associated with opposite epigenetic modifications on similar genes, wherein resilience is associated with increased methylation and decreased expression of CRH and vulnerability is associated with demethylation and increased expression of CRH and *fkbp5* in response to chronic stress (Elliott et al., 2010).

Epigenetic writers themselves are also candidates for mediating risk and resilience, in part through managing an appropriate balance between epigenetic repressors and epigenetic activators (Zovkic et al., 2013). Under baseline conditions, epigenetic repressors, such as HDACs, keep gene expression in check, whereas neuronal signaling increases gene expression in part by relieving such inhibition (Lattal et al., 2007). This repressive action of HDACs and other molecules has been termed the molecular break pad, reflecting the involvement of these molecules in regulating the magnitude and the duration of gene expression (Lattal and Wood, 2013). For example, reduction of HDAC3 expression prolongs the expression of immediate early genes involved in learning and memory (McQuown and Wood, 2011; McQuown et al., 2011) and HDAC inhibition or knockdown enhances fear learning in rodents (Levenson et al., 2004; Guan et al., 2009; Graff and Tsai, 2011; Lesburgueres et al., 2011). An implication of these studies is that a disrupted balance favoring activators over repressors may promote PTSD vulnerability by increasing the intensity of the traumatic memory, which is consistent with reports of reduced HDAC2 expression in the post-mortem brains of human patients suffering from depression (Sun et al., 2013).

However, not all HDAC subtypes are memory repressors. For example, sirtuins are class III HDACs that have been implicated in improved cognition (Kim et al., 2007) and hippocampal overexpression of HDAC1 was recently associated with improved extinction of fear memory (Bahari-Javan et al., 2012). Although sirtuins tend to be regulated by a distinct class of compounds, many pharmacological HDACi influence a broad range of HDACs, making it difficult to associate behavioral outcomes with specific HDAC subtypes. For example, although chronic social defeat was found to selectively increase the expression of the memory repressor HDAC2, treatment with the HDACi mass spectrometry (MS)-275 had an anti-depressant effect in these rodents (Guan et al., 2009; Covington et al., 2011b). This discrepancy may in part be associated with the relative specificity of MS-275 for HDAC1 over other HDACs, including HDAC2 (reviewed in Grayson and Guidotti, 2013). Similarly, TSA is an HDACi that actually increases HDAC1 expression in cell culture (Ajamian et al., 2004) and given the positive role of this HDAC on fear extinction (Bahari-Javan et al., 2012), TSA-induced enhancement of cognitive function may at least partly be a reflection of enhanced expression of HDAC1.

In addition to HDACs, DNMTs are emerging as another class of multifunctional epigenetic regulators with distinct modifications in PTSD, as evidenced by hypomethylation of DNMT3B gene and hypermethylation of the DNMT3L gene (Uddin et al., 2010). HDACs and DNMTs are only few of many epigenetic regulators involved in mediating the balance between activators and repressors and a thorough understanding of the potential shifts in their balance will require the consideration of these enzymes in the context of other epigenetic mediators, including histone acetylases, methylases, and demethylases, TET proteins, and other enzymes that directly mediate epigenetic modifications. Complementing these studies with genome-wide changes in gene expression will provide valuable information regarding the effects of specific modifications on genes associated with risk and resilience.

## Cognitive Aspects of PTSD: Cause and Outcome

In addition to deregulation of the stress response, PTSD involves the development of strong cognitive associations between the stressful/fear-inducing event and the context or cue in which the event took place. This aspect is strongly tied into PTSD symptom expression, in that the stimuli or cues that are similar to those associated with trauma can on their own trigger re-experiencing of traumatic memories long after the event has passed (Ehlers et al., 2004). Epigenetic mechanisms are particularly relevant in this regard because of their role in the persistent stabilization of memory for transient fear-inducing stimuli (Zovkic et al., 2013). Existing literature on the epigenetics of fear learning has been conducted primarily in the context of cognition, but the use of fear conditioning models in PTSD makes these studies directly relevant to the disorder. Epigenetic modifications in fear learning have been extensively reviewed in a recent paper (Zovkic and Sweatt, 2013) and will be discussed here only briefly to illustrate the current state of understanding and the future directions required to advance the application of these models to mechanistic and clinical studies of PTSD.

A major advantage of animal models of fear learning is the ability to assess fear expression at defined time points after the fear-inducing stimulus to track the molecular mechanisms involved in the establishment and perpetuation of fear memory. Assessment of fear learning shortly after shock (2 h) best reflects the initial response to trauma, but this is only an index of short term memory and is not well suited for assessing persistent symptoms of PTSD, particularly given the lack of an initial emotional response in many patients with PTSD (Friedman et al., 2011a,b). However, this is a critical time point for evaluating epigenetic mechanisms of fear memory consolidation that are required for stabilizing the memory over time (Zovkic et al., 2013). Molecular assessment at this time point may reveal different patterns or intensities of gene expression and associated epigenetic modifications in vulnerable and resilient individuals that can inform efforts to identify mechanisms for pathological fear formation. Consolidation of cued and contextual fear conditioning is mediated by epigenetic modifications in the amygdala, whereas epigenetic modifications in the hippocampus are specifically involved in mediating contextual fear learning (Levenson et al., 2004; Lubin and Sweatt, 2007; Miller et al., 2008; Monsey et al., 2011). Consolidation of hippocampus-dependent fear memory is typically evaluated 24 h after training, whereas consolidation of remote memory becomes evident at least 7 days after training, when the memory from the hippocampus has been “downloaded” to the cortex for maintenance (Frankland et al., 2004; Ding et al., 2008). A focus on the remote time point is especially relevant because it provides the best approximation of the persistent and intrusive memories seen in patients with PTSD. Given the well-defined nature of the brain regions involved in fear consolidation and maintenance, the straight-forward behavioral read out of fear learning (i.e., freezing), and the ease with which stimulus (i.e., shock) intensity can be modified, this model provides an excellent platform on which to build our understanding of epigenetic mechanisms of PTSD. For a full review of epigenetic mechanisms of fear learning and their implications for PTSD, please refer to a recent review from our lab (Zovkic and Sweatt, 2013).

### Memory consolidation

It is now well established that epigenetic mechanisms are essential mediators of fear memory consolidation. Histone acetylation, phosphorylation, and methylation play a critical role in hippocampus- and amygdala-dependent consolidation across a range of conditioning paradigms (Chwang et al., 2006; Levenson et al., 2006; Lattal et al., 2007; Lubin and Sweatt, 2007; Bredy and Barad, 2008; Lubin et al., 2008; Miller et al., 2008; Maddox and Schafe, 2011; Monsey et al., 2011; Stafford et al., 2012) and interact with DNA methylation to mediate memory formation (Miller et al., 2008; Maddox and Schafe, 2011). Pharmacological or genetic interference with DNA methylation impairs fear memory (Miller and Sweatt, 2007; Miller et al., 2008, 2010; Feng et al., 2010), whereas enhancement of histone acetylation through HDAC inhibition enhances memory formation through reduced threshold for memory formation (Levenson et al., 2004; Bredy and Barad, 2008; Miller et al., 2008; Graff and Tsai, 2011; Maddox and Schafe, 2011; Monsey et al., 2011). Similarly, knocking out *gadd45*, which is involved in DNA demethylation, improves memory performance (Sultan et al., 2012), indicating that memory may be enhanced through a paradoxical upregulation of both DNA methylation and histone acetylation. Although a number of explanations have been offered for this paradox (Zovkic et al., 2013), recent advances implicating DNA methylation in gene activation may also contribute to a resolution. Nevertheless, the observation of improved memory with HDACi indicates that individuals with lower levels of HDACs and higher levels of enzymes involved in DNA demethylation may be more sensitive to developing fear responses that are disproportional to the traumatic event. This proposition is particularly relevant in the context of the molecular breakpad hypothesis, in which synaptic activity overrides the normally repressive effects of HDACs on transcription (Lattal and Wood, 2013), in that lower levels of HDACs may allow subthreshold levels of synaptic activity to produce a strong memory. Thus, an essential aim of PTSD research must be to fully characterize the relationship between different epigenetic enzymes and PTSD, as they relate to the consolidation of traumatic experiences. Advances have already been made in studies of group differences under baseline conditions in studies of human patients, which found reduced levels of HDAC2 and altered methylation of DNMT3L genes in PTSD (Uddin et al., 2010; Sun et al., 2013).

### Memory maintenance

Hippocampus-dependent memory consolidation occurs within hours of fear conditioning, when the memory begins a gradual process of downloading to the cortex for maintenance (Frankland and Bontempi, 2005). Epigenetic mechanisms have been implicated in the initial memory transfer process in which histone modifications in the cortex serve as a memory transfer tag (Lesburgueres et al., 2011) and as a persistent mark for memory maintenance (Graff et al., 2012). In contrast to the transient changes in DNA methylation observed in the hippocampus, fear conditioning is associated with a persistent increase in DNA methylation and decreased expression of the gene coding for the memory suppressor calcineurin up to 30 days after fear conditioning (Miller et al., 2010). DNA methylation is critical for memory maintenance at this time point, such that blocking DNA methylation immediately before a 30 day memory test impairs recall (Miller et al., 2010). The role of DNA methylation in memory persistence is of particular relevance to PTSD given the persistent re-experiencing of the traumatic event, indicating that manipulation of the epigenome at appropriate time points may allow for interference with previously established traumatic memories (see Zovkic and Sweatt, 2013 for an expanded discussion of therapeutic implications).

### Memory reconsolidation and extinction

Persistent and intrusive memory recall is the foremost feature of PTSD in humans (Orr et al., 1993). Studies of molecular events associated with recall are critical for understanding the molecular mechanisms that underlie the strength of the pathological memory and for identifying potential treatments. Memory recall is an active process that renders the memory labile and employs molecular mechanisms to re-stabilize the original memory (Bredy and Barad, 2008; McKenzie and Eichenbaum, 2011; Pitman et al., 2011; Lattal and Wood, 2013). From a treatment perspective, interference with reconsolidation provides an opportunity to destabilize and even erase the original traumatic memory and this avenue is being actively pursued in patients with PTSD (Pitman, 2011). A recalled memory also becomes subject to a second active process termed extinction. Extinction occurs when a previously learned association is altered by new information, such as the safety of a conditioned stimulus in which a shock had previously occurred (Bouton, 2004). In contrast to disrupted reconsolidation, which effectively promotes forgetting, enhancing extinction allows for new learning in which the fear-inducing component of the memory can be selectively disrupted while leaving the initial memory relatively intact (Lattal and Wood, 2013). PTSD is often considered to be a disorder of extinction (Koenigs and Grafman, 2009) and many studies with rodents have found impaired extinction in at-risk individuals. For example, low LG and maternally separated offspring exhibit enhanced fear conditioning and impaired extinction (Champagne et al., 2008; Bagot et al., 2009; Callaghan and Richardson, 2011), whereas others associated brief maternal separation (15 min) with impaired fear extinction in spite of similar rates of initial conditioning (Wilber et al., 2007, 2009; Stevenson et al., 2009). Deficits in extinction have also been associated with the serotonin transporter in rodents (Wellman et al., 2007; Narayanan et al., 2011) and in people (Hartley et al., 2012), indicating that genetic risk for PTSD may be partly mediated by impaired fear extinction.

A major challenge in applying extinction and reconsolidation as a long-term treatment is the relative transience of extinction relative to the original learning event and the requirement for pharmacological intervention for robust interference with reconsolidation (Pitman, 2011). However, recent studies in rodents suggest that extinction may be an effective treatment strategy when combined with manipulations of the epigenome that can stabilize extinction learning over time (Lattal et al., 2007; Bredy and Barad, 2008; Lattal and Wood, 2013). Specifically, administration of HDACi promotes extinction of fear learning and robust extinction of cocaine preference that is resistant to subsequent reinstatement by cocaine (Malvaez et al., 2010), indicating that HDAC inhibition combined with extinction may promote adaptive learning that can combat deleterious adaptations. The effects of HDACi on extinction are dependent primarily on the HDAC1 subtype (Bahari-Javan et al., 2012) and are most effective in the infralimbic cortex (Stafford et al., 2012), which is consistent with evidence that HATs mediate extinction specifically in this brain region (Marek et al., 2011; Wei et al., 2012). This regional specificity is of functional relevance because it selectively alters extinction without impacting initial learning (Wei et al., 2012) and of translational relevance because lesions of the ventromedial prefrontal cortex (vmPFC) impair long-term extinction in people (Quirk et al., 2000) and reduced vmPFC activity has been reported in patients with PTSD (Koenigs and Grafman, 2009). These studies point to a dissociation between fear learning and fear extinction, wherein deficits in extinction are a critical characteristic of PTSD (Schnurr and Friedman, 2008) and may be more relevant to treatment because of the temporal gap between trauma and treatment onset. This temporal and regional difference also has implications for HDAC treatment, in that HDAC inhibition around the time of trauma exposure increases fear memory, whereas HDAC inhibition in association with fear extinction enhances the extinction of fear memory. This distinction is important to keep in mind when developing therapies for PTSD.

Extinction, in the form of exposure therapy, is heavily utilized treating PTSD (Schnurr and Friedman, 2008) and some effort has been made toward implementing reconsolidation based approaches in treatment (Pitman, 2011). However, more work is needed to identify the best conditions under which behavioral therapies are most effective when combined with pharmacological therapies. Clinical studies suggest that contextual parameters utilized in extinction and reconsolidation based therapies are critical for treatment efficacy, in that similarity between treatment conditions and those associated with the initial trauma has a major impact on treatment outcomes (Pitman, 2011). In humans and in animal models, this concept is typically investigated under the umbrella of fear generalization, whereby neutral cues can elicit fear responses based on their degree of similarity to cues or contexts associated with traumatic stimuli (Dunsmoor et al., 2009; Iordanova and Honey, 2012), but we are not aware of studies that have directly investigated epigenetic mechanisms in relation to fear generalization. In humans, there is increased use of virtual reality-based therapies to better reconstruct traumatic scenarios (Davis et al., 2006a,b) and similar approaches can be used to predict traumatic scenarios for soldiers before going into combat. Nevertheless, it is still necessary for animal studies to combine a thorough behavioral assessment with epigenetically based pharmacological interventions to identify the appropriate conditions for subsequent testing in human patients.

## Epigenetics in Diagnosis and Intervention

Epigenetic modifications may be particularly strong candidates for use in assessing at-risk individuals for early intervention, as well as for PTSD treatment through epigenetically based therapies. An important first step toward such use of epigenetics involves the establishment of “epigenetic signatures” that serve as diagnostic markers and help identify targets for directed treatment. The idea of an epigenetic signature is driven by the assumption that PTSD is associated with consistent alterations in the epigenome across individuals and that these modifications can be used as markers of risk, resilience, and symptom severity. We have discussed much of the substantial progress that has been made toward identifying common epigenetic modifications in at-risk and resilient populations (see discussion of gene-environment-epigenome interactions above) and systematic investigations of these modifications can be of tremendous importance for directing early intervention (Uddin et al., 2010). Indeed, studies in animal models have exhibited encouraging results regarding the reversal of risk factors and cognitive deficits through enrichment and epigenetic intervention in juvenile and adult rodents (e.g., Francis et al., 2002; Fischer et al., 2007), indicating that early detection of risk may be key for PTSD prevention. Epigenetic markers will also be useful in PTSD diagnosis and in tracking therapeutic efficacy after trauma.

Although it is unlikely that any two individuals will have identical epigenetic profiles in response to trauma, it is conceivable that a substantial amount of overlap in essential genes will be found across patients, as evidenced by reports of altered *DNMT*, *FKBP5*, *SLC6A4*, and *GR* methylation reviewed above. Moreover, recent studies have demonstrated similar levels of DNA methylation of *SLC6A4* and *FKBP5* genes in the blood and the brain (Suomi, 2011; Klengel et al., 2013), indicating that the epigenetic status of certain genes in the brain may be indicated by changes in the blood. However, differences in function between immune cells and neurons must be taken into account, as early life stressors are known to modify immune cell function (Lam et al., 2012) and immune system alterations are commonly observed in PTSD (Smith et al., 2011). The degree to which concordance between brain and blood is replicable and applicable to other genes remains to be determined, but such concordance is less relevant for many clinical purposes than the ability of peripheral markers to reliably predict PTSD symptoms.

Nevertheless, tracking changes in pre- and post-treatment epigenetic profiles would be particularly useful for identifying genes that are modified by behavioral and epigenetic interventions as a first step in developing targeted therapies. In addition, it may be possible to identify the changes that are associated with persistent positive outcomes and to use this information to tailor more effective therapies. Indeed, an extinction-specific role of HDAC1 has already been identified for fear learning and a library of such markers would provide a valuable tool for designing epigenetic therapies to specifically enhance the efficacy of corresponding behavioral therapies. The efficacy of epigenetic intervention is evidenced by the fact that anti-depressant drugs produce epigenetic modifications (Tsankova et al., 2004, 2006) and that epigenetic drugs have anti-depressant effects (Covington et al., 2011b).

Epigenetic therapies, including HDACi, may be particularly strong candidates for PTSD treatment because they do not target a single neurotransmitter system and often exhibit therapeutic or behavioral effects only under appropriate signaling conditions (Roozendaal et al., 2010; Graff and Tsai, 2011). These pharmacological features are relevant to many psychiatric disorders, including PTSD, which exhibit deregulation of multiple neurotransmitter and signaling molecules (e.g., serotonin, NPY, GR, Fkpb5, BDNF, and CRH) and are typically treated by drugs such as fluoxetine that are targeted against specific neurotransmitter systems (Steckler and Risbrough, 2012). It is now evident that up- and down-regulation of the various deregulated molecules is at least partly mediated by altered DNA methylation and histone acetylation (Costa et al., 2003; Dong et al., 2007; Kinnally et al., 2011; Koenen et al., 2011; Klengel et al., 2013), indicating that epigenetic drugs may stabilize function across a range of disrupted molecules. For example, upregulation of *FKBP5* in high-risk individuals is associated with reduced methylation of the GRE, which is demethylated by exposure to glucocorticoids during early development (Klengel et al., 2013). Similarly, reduced levels of reelin and 67-kDA glutamate decarboxylase (GAD_67_) have been attributed to DNA hypermethylation in mice (Costa et al., 2003; Dong et al., 2007).

On the flip side, many of the molecules we are discussing also alter gene expression through downstream effects on the epigenome and some evidence suggests that the bidirectional relationship across levels of regulation is a key driver or appropriate and stimulus-specific effects on behavior. For example, PKA-pCREB/CBP signaling and GR receptor activation drive changes in histone acetylation and hippocampus-dependent learning (Levenson et al., 2004; Roozendaal et al., 2010; Maddox and Schafe, 2011; Monsey et al., 2011), but HDACi could only enhance learning in the presence of GR activation (Roozendaal et al., 2010), indicating that increasing histone acetylation exerts behavioral effects only in the presence of appropriate signaling.

In addition to driving gene expression in mental disorders, epigenetic drugs may also normalize deregulated gene expression. For example, reduced gene expression and increased DNA methylation in schizophrenia were reversed by treatment with HDACi and this effect was associated with an improvement in psychotic symptoms (Costa et al., 2003; Tremolizzo et al., 2005a; Dong et al., 2007). Similarly, positive outcomes were reported for the HDACi valproic acid when given in conjunction with antipsychotics (Wassef et al., 2000, 2001; Grayson et al., 2010) or anti-depressants (Schroeder et al., 2007). Additionally, studies in rodents reported enhanced extinction of fear (Lattal et al., 2007; Bredy and Barad, 2008) and reduced depression (Covington et al., 2011b) in response to HDACi treatment, indicating that these drugs may be efficacious through effects on cognitive and affective processes. Even treatments that do not directly manipulate the epigenome produce epigenetic changes, with behavioral enrichment improving cognitive deficits through similar effects on chromatin regulation as HDACi (Fischer et al., 2007). Thus, a combination of environmental and pharmacological treatments may provide strongest candidates for early intervention or treatment. However, it is important to caution that outcomes associated with HDAC treatment in humans have been mixed (Narayan and Dragunow, 2010) and may be partly attributed to administration of adjunct therapies and the stage of the disease at the time of treatment (Tsai review). The development of more specific drugs and under appropriate conditions will be essential for improving the clinical efficacy of epigenetic therapies.

A number of issues must be addressed in designing epigenetic drugs with greater efficacy, including improved blood-brain-barrier (BBB) permeability and increased specificity for brain regions and HDAC subtypes (Grayson et al., 2010). HDACs are differentially distributed throughout the brain (Broide et al., 2007) and a growing number of studies point to distinct effects of specific HDAC subtypes in distinct brain regions and behavioral phenomena (Kim et al., 2007; Bahari-Javan et al., 2012; Sun et al., 2013). There is already a range of drugs that have different levels of regional and subtype specificity, with one study reporting that systemic injections of the BBB-permeable MS-275 (an HDACi selective for HDAC1 over HDAC3 and 8) selectively enhanced H3 acetylation in the frontal cortex and the hippocampus, but not the striatum (Simonini et al., 2006). In that study, MS-275 was more potent than valproic acid, which induced similar changes in acetylation across brain regions (Simonini et al., 2006). With more studies demonstrating HDAC-subtype specific effects on neural function and behavior (Kim et al., 2007; Bahari-Javan et al., 2012; Hanson et al., 2013), drugs that can be systemically administered and produce selective effects will be critical for targeted treatments. Interestingly, studies of human T23 bladder and MDA breast carcinoma, as well as HL60 cells, found a similar proportion of genes to be up- and down-regulated in response to treatment with HDACi (Glaser et al., 2003; Halsall et al., 2012), indicating that the effects of HDACi are not exclusively activating. Indeed, a high level of complexity and balance between memory activators and repressors necessitates that some genes are turned off in order for others to be effectively turned on (Zovkic et al., 2013).

Discussions of epigenetic treatments rightfully caution about the potential for wide-spread and non-specific effects on gene expression. However, microarray analysis of changes in hippocampal gene expression in response to l-methionine treatment only affected 1% of all the genes (Weaver et al., 2006), indicating that manipulations of epigenetic precursors and enzymes may produce surprisingly specific outcomes. Similarly, treatment of HL60 cells with three commonly used HDACi (VPA, TSA, SAHA) altered the expression of approximately 9% of genes, with only minor increases in histone acetylation observed at gene promoters (Halsall et al., 2012).

Moreover, manipulations of HDACs result in at least partly discriminant changes in acetylation, with HDAC1 having no effect on AcH3K14 and AcH4K5 (Bahari-Javan et al., 2012) and MS-275 altering AcH3K9 without affecting H4K12 (Simonini et al., 2006; Peleg et al., 2010). In addition to gene- and residue-specific effects, HDAC2 selectively enhances inhibitory synaptic function in the CA1 (Hanson et al., 2013), indicating that there is potential for specificity among epigenetic therapies. Much work is still needed to improve target specificity and the mechanisms of HDACi actions are not always clear, given the potential for the disruption of repressive complexes and effects on non-histone targets, which can be difficult to tease apart from effects of these drugs on histone acetylation (Zovkic et al., 2013). Thus, the development of increasingly specific therapies and a full characterization of the effects of existing therapies are important for developing drugs that can influence specific types of neural activity in selected brain regions.

## Future Directions

In the present review, we focused heavily on developmental and genetic factors that predispose or protect individuals from PTSD. In many of these studies, the degree to which a manipulation is adaptive in adulthood is determined by reactivity to acute stressors outside of the context of PTSD. While these studies provide valuable insights into variable responses to trauma, a thorough assessment of persistent symptoms over prolonged periods of time is required to better model the lasting and intrusive nature of PTSD in people. Fear conditioning is one example of a model that is amenable to testing at different time points that can be used to characterize epigenetic changes that extend beyond the initial response to trauma and include trauma maintenance (see Tables 1 and 2 for summary of epigenetic modifications in fear conditioning and PTSD models). It will also be important to combine developmental models with models of PTSD that sort animals according to symptom development, severity, and persistence. This will be especially useful in distinguishing between early life experiences acting as stress inoculators vs. risk inducers in relation to adult risk and resilience. Ideally, early life manipulations will be combined with genetic models to reflect the gene-environment interactions identified in human risk populations. It is important to note that the rapid pace at which novel techniques are developed poses an opportunity and a challenge for conducting cutting edge research that can shed new light on our understanding of PTSD and psychiatric disorders. Most rapid advancements are particularly evident for techniques differentiating between DNA methylation and hydroxymethylation. Given the role of 5hmC in DNA demethylation and the distinct association of each modification with gene regulation, we have included a summary of new approaches to facilitate their application in studies of PTSD (see **Box 1**).

**Table 1:** **A summary. of epigenetic modifications reported in rodent models of fear conditioning**.

Epigenetic modification measured	Gene	Brain region	Effect	Reference
**MEMORY CONSOLIDATION (30 min–2 h AFTER FEAR CONDITIONING)**				
H3 acetylation	Global	CA1	↑	Chwang et al. (2006), Levenson et al. (2004), Miller et al. (2008)
	Bdnf IV promoter	CA1	↑	Lubin et al. (2008)
		Hippocampus	↑	Takei et al. (2011)
	Homer 1 promoter	Hippocampus	↑	Mahan et al. (2012)
	Global	Lateral amygdala	↑	Monsey et al. (2011), Maddox et al. (2013)
H3 phosphorylation	Global	CA1	↑	Chwang et al. (2006)
H3 phosphoacetylation	Global	CA1	↑	Chwang et al. (2006)
H3K9me2	Global	Entorhinal cortex	↑	Gupta-Agarwal et al. (2012)
H3K4me3	Global	CA1	↑	Gupta et al. (2010), Gupta-Agarwal et al. (2012)
	*zif268* promoter	CA1	↑	Gupta et al. (2010)
	*BDNF I*	CA1	↑	Gupta et al. (2010)
	Homer 1 promoter	Amygdala	↓	Mahan et al. (2012)
DNA methylation	PP1	CA1	↑	Miller and Sweatt (2007)
	Reelin		↓	Miller and Sweatt (2007)
	Bdnf		↓	Lubin et al. (2008)
	zif268		↑	Gupta et al. (2010)
**MEMORY MAINTENANCE (7–30 DAYS)**				
DNA methylation	Calcineurin	PFC	↑	Miller et al. (2010)

**Table 2:** **A summary of epigenetic modifications in human and animal models of PTSD**.

Species/model	Gene(s) of interest	Major findings	Reference
**CANDIDATE-GENE STUDIES**			
Human	*ADCYAP1*, *ADCYAP1R1*	PTSD symptoms correlated with *Adcyap1r1* locus in women	Ressler et al. (2011)
Rat – predator odor + social instability	*Bdnf*	↑ Exon IV methylation in dorsal DG and CA1, ↓exon IV methylation in ventral CA3, ↓exon IV mRNA in both dorsal and ventral CA1	Roth et al. (2011)
Human	*SLC6A4*	Controlling for genotype, *SLC6A4* methylation modified the effect of PTEs on PTSD: ↓ *SLC6A4* promoter methylation associated with ↑PTSD risk; ↑*SLC6A4* promoter methylation was protective against PTSD	Koenen et al. (2011)
Human	*SLC6A3*	↑ *SLC6A3* promoter methylation associated with ↑risk of lifetime PTSD in 9R allele carriers	Chang et al. (2012)
Human	*COMT*	*COMT* Met/Met genotype interacted with CpG methylation in mediating impaired fear inhibition in PTSD patients	Norrholm et al. (2013)
Human	*FKBP5*	GC exposure was associated with ↑ *FKBP5* GRE demethylation and ↑*FKBP5* expression in carriers of the risk compared with the protective allele	Klengel et al. (2013)
**GENOME-WIDE/LARGE SCALE STUDIES**			
Human	Genes involved in immunity, neurogenesis, the startle response, *DNMT3B*, *DNMT3L*, imprinted genes: *NDN, MAGEL2, ATP10A*	PTSD was associated with: (1) ↑ methylation of *DNMT3B*, ↓methylation of *DNMT3L*; (2) degulated methylation of genes involved in Prader–Willi and Angelman syndromes; (3) methylation profiles suggesting upregulation of immune-related genes and downregulation of genes involved in neurogenesis and the startle response	Uddin et al. (2010)
Rat – predator odor	*Dlgap2*, *Dll3*, *Pkc*η, *Rps6kb2*	Of the four differentially methylated genes identified, *Dlgap2* was associated with a change in mRNA expression. ↓ intragenic methylation associated with ↓hippocampal mRNA expression	Chertkow-Deutsher et al. (2010)
Human	*TPR*, *CLEC9A*, *APC5*, *ANXA2*, *TLR8*, *BDNF*, *CXCL1*, immune-related genes	PTSD associated with: (1) ↓ methylation of *TPR* and *ANXA2* and ↑methylation of *CLEC9A*, *APC5*, *TLR8* in PTSD; (2) ↑methylation of BDNF and CXCL1; (3) 19 of 54 of the differentially methylated immune-related genes examined in Uddin et al. (2010)	Smith et al. (2011)
**OTHER**			
Human	33 loci previously associated with PTSD	Only *MAN2C1* showed evidence of interaction with PTE no. in PTSD risk: ↑ *MAN2C1* methylation interacted with ↑no. of PTEs to ↑PTSD risk	Uddin et al. (2011)
Human	Repetitive elements: *LINE-1*, *Alu*	In US military service members recently deployed to Afghanistan or Iraq: LINE-1 was hypomethylated in PTSD cases vs. control post-deployment. *Alu* was hypermethylated in PTSD cases vs. control pre-deployment	Rusiecki et al. (2012)

*PTE, potentially traumatic event; TLS, total life stress; ADCYAP1, adenylate cyclase-activating polypeptide 1 (pituitary); ADCYAP1R1, adenylate cyclase-activating polypeptide 1 (pituitary) receptor type I; Bdnf, brain-derived neurotrophic factor; SLC6A4, solute carrier family 6 (neurotransmitter transporter, serotonin), member; SLC6A3, solute carrier family 6 (neurotransmitter transporter, dopamine), member; COMT, catechol-*O*-methyltransferase; FKBP5, FK506 binding protein 5; DNMT3B, DNA methyltransferase-3B; DNMT3L, DNA methyltransferase-3L; NDN, necdin, melanoma antigen (MAGE) family member; MAGEL2, MAGE-like 2; ATP10A, ATPase, class V, type 10A; Dlgap2, disks large-associated protein 2; Dll3, delta-like 3; Pkcη, protein kinase C η; Rps6kb2, ribosomal protein S6 kinase polypeptide 2; TPR, translocated promoter region; CLEC9A, C-type lectin domain family 9, member A; APC5, acid phosphatase 5, tartrate resistant; ANXA2, annexin A2; TLR8, toll-like receptor 8; CXCL1, chemokine (C-X-C motif) ligand 1; MAN2C1, mannosidase, alpha, class 2c, member 1*.

In addition to affecting gene expression through the direct interaction with transcriptional machinery, DNA methylation is increasingly being recognized as an incredibly complex regulator of neuronal function and metaplasticity (Baker-Andresen et al., 2013). For example, *de novo* DNA methylation was shown to prevent RNA polymerase stalling, thus providing a mechanism by which DNA methylation can regulate alternative splicing (Shukla et al., 2011). The long-term regulation of alternative splicing by DNA methylation may affect the responsivity of neurons to future stimuli (Baker-Andresen et al., 2013); thus investigations into the epigenetic regulation of alternative splicing are warranted in delineating factors that convey resilience to PTSD. Additionally, DNA methylation has been implicated in the regulation of retrotransposons, such as the positioning of long interspersed nuclear elements-1 (LINE-1) (Muotri et al., 2010). This is important, as the insertion of LINE-1 into various genomic regions may increase gene length, which is associated with decreased efficiency of expression (Castillo-Davis et al., 2002). Interestingly, a recent report demonstrated that a post-deployment diagnosis of PTSD was associated with hypomethylation of LINE-1, suggesting that methylation of these elements may play a role in mediated risk or resilience (Rusiecki et al., 2012). The existence of 5hmc and of distinct cofactors that interact with 5mc and 5hmc (Spruijt et al., 2013) further complicates the interpretation and calls for the use of techniques that distinguish between these modifications (see **Box 1**). Such approaches will be critical for identifying epigenetic signatures that differentiate between risk and resilience.

Finally, although women are more likely to develop PTSD than men, mot rodent studies have been conducted only in males (Mulchahey et al., 2001; Pope et al., 2003). Thus, a greater focus on females is warranted and may help us understand the basis for relative resilience in men, with testosterone providing one possible candidate for resilience (Russo et al., 2012). Some existing evidence suggests that sex differences are will be relevant for understanding all aspects of PTSD, with prenatal stress producing altered levels of DNMT1 expression, hippocampal GR methylation, and hypothalamic CRH methylation in adult male, but not female, offspring (Mueller and Bale, 2008). In addition, evidence from rodent studies suggests that sex differences vary for distinct components of PTSD, with females exhibiting vulnerability in tests of motivation and affect, while exhibiting resilience in non-stressful cognitive tasks (Luine, 2002).

## Conclusion

In this review, we have demonstrated that epigenetic mechanisms are fundamental regulators of predisposition and resilience to PTSD, primarily through mediating gene-environment interactions during sensitive periods of development. Epigenetic mechanisms are also essential mediators of proximal causes of PTSD because of their role in stabilizing persistent outcomes of transient traumatic events. Although much progress has been made toward identifying epigenetic contributions to PTSD, ongoing shifts in our fundamental understanding of epigenetic modifications and their function call for the use of new technologies to clarify the role of these modifications in psychiatric disorders. As our appreciation of the complex roles of epigenetic mechanisms increases, so will our ability to create new and effective interventions for this incredibly complex and devastating human disease.

## Conflict of Interest Statement

The authors declare that the research was conducted in the absence of any commercial or financial relationships that could be construed as a potential conflict of interest.
